# Successful laparotomic ethanol ablation for an adrenal tumour in a dog

**DOI:** 10.1002/vms3.70020

**Published:** 2024-09-17

**Authors:** Shimon Furusato, Eriko Kondo, Yu Tamura, Yu Tsuyama

**Affiliations:** ^1^ Shinagawa WAF Animal Hospital Tokyo Japan; ^2^ Present address: JASMINE Veterinary Cardiovascular Medical Center Kanagawa Japan; ^3^ Nagaya Animal Medical Center Aichi Japan

**Keywords:** adrenalectomy, adrenal gland neoplasm, adrenal insufficiency, adrenocortical hyperfunction, dogs, ethanol

## Abstract

Adrenalectomy is the gold standard for canine adrenal tumours, but not always recommended due to patient age, underlying conditions and perioperative mortality. Ethanol ablation is an alternative in human medicine for poor surgical candidates. A 13‐year‐old neutered female toy‐poodle with hypercortisolism presented with severe haematuria. Ultrasonography revealed left adrenal and right kidney tumours. Due to high surgical risk, simultaneous laparotomic right nephroureterectomy and ethanol ablation of the left adrenal tumour were performed. Post‐ethanol injection complications included transient hypertension and arrhythmia, which resolved spontaneously. The adrenal tumour size decreased within 2.5 months, and cortisol levels normalised within 8 days, remaining stable for 12 months. No hypercortisolism signs were observed without trilostane until death from renal insufficiency. Autopsy showed that the ablated left adrenal gland was an adrenocortical tumour and had shrunk. Ethanol ablation may be a feasible alternative to adrenalectomy for high‐risk canine patients.

## Graphical abstract

Adrenalectomy is the gold standard for canine adrenal tumours, but it is not always feasible due to surgical risks. We successfully conducted simultaneous laparotomic right nephroureterectomy and ethanol ablation of the left adrenal tumour. The adrenal tumour size decreased, cortisol levels normalised and no signs of hypercortisolism were observed for 12 months. Ethanol ablation of adrenal tumours may be a viable alternative for high‐risk canine patients.

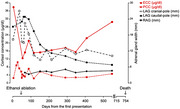

## 1 INTRODUCTION

Hypercortisolism is a common endocrine disease in middle‐aged and older dogs (Behrend, 2014). Eighty‐five percent of spontaneous canine hypercortisolism cases are pituitary dependent, and the remaining 15% are due to adrenal tumours (Herrtage & Ramsey, 2012). Although adrenalectomy is considered the gold standard treatment for adrenocortical tumours, technical challenges and postoperative complications, including bleeding and life‐threatening acute adrenal insufficiency (Di Dalmazi et al., 2014; Raman et al., 2019; Shen et al., 2006) are concerning; moreover, it carries 8%–24% perioperative mortality rates (Anderson et al., 2001; Cavalcanti et al., 2020; Knight et al., 2019; Kyles et al., 2003; Massari et al., 2011; Mayhew et al., 2019; Merlin & Veres‐Nyéki, 2019; Schwartz et al., 2008). Medicinal treatments, such as mitotane and trilostane, are alternatives to adrenalectomy for high‐risk surgical patients or when owners decline surgical treatment. However, these medications require lifelong frequent administration, are not curative, and may also cause adverse gastrointestinal and neurological effects and adrenal insufficiency (Arenas et al., 2014; Kintzer & Peterson, 1994). Furthermore, radiotherapy has emerged as a less invasive alternative to adrenalectomy, but has some disadvantages, including high cost, limited availability of facilities and the need for multiple anaesthesia (Dolera et al., 2016; Nolan & Gieger, 2024).

In humans, ultrasonography (US)‐ or computed tomography (CT)‐guided percutaneous chemical ablation has been established as a treatment modality for adrenal tumours in high‐risk surgical patients (Chang et al., 2012; Maki et al., 2000; Minowada et al., 2003, Rossi et al., 1995; Wang et al., 2003; Xiao et al., 2008). Ethanol ablation, a type of chemical ablation, has the advantages of being simple, inexpensive and less invasive, and its effect persists for a long period (6–54 months), although multiple sessions may be required (Chang et al., 2012; Maki et al., 2000; Rossi et al., 1995; Wang et al., 2003; Xiao et al., 2008). Ethanol ablation is an accepted modality for treating primary hyperparathyroidism in veterinary clinical practice (Guttin et al., 2015; Long et al., 1999; Rasor et al., 2007; Riehl et al., 2019); however, there are no reports of ethanol ablation for functional adrenal tumours in veterinary medicine. Here, we report the clinical course of laparotomic ethanol ablation for a functional adrenal tumour in a high‐risk surgical canine patient.

## 2 CASE DESCRIPTION

A 13‐year‐old female neutered toy‐poodle dog weighing 5 kg presented with severe haematuria, which was not resolved after antibiotic (orbifloxacin, 4 mg/kg, q24h) and non‐steroidal anti‐inflammatory drug (unknown drug name; day 1). The dog was previously diagnosed myxomatous mitral valve degeneration (MMVD) stage C (Keene et al., 2019) and cutaneous pruritus. The dog had been treated with pimobendan, benazepril, furosemide and oclacitinib previously at another clinic. The dog showed excessive panting, pot‐bellied appearance, polyuria and polydipsia. Physical examination revealed symmetrical trunk alopecia, systemic superficial pyoderma and a systolic heart murmur (grade 3/6). Urinalysis revealed abundant red blood cells, epithelial cells, cellular casts and slight struvite crystals in urine sediment. Urine specific gravity was 1.025. Abdominal US examination (Blind information) revealed a mass in the right kidney (41.2 mm × 30.9 mm) and a left adrenal gland (LAG) mass (21.1 mm in width × 38.2 mm in length) with an upper limit of reference interval (RI): 5.1 mm in maximum width (Melián et al., 2021). The right adrenal gland (RAG) was difficult to delineate because the right kidney mass was disturbed. The liver had a homogeneous parenchymal echogenicity with slightly blunt edges and no focal lesions were observed on ultrasound. No abnormalities were observed in the other abdominal organs. Echocardiography and thoracic and abdominal radiography revealed left atrial enlargement, cardiomegaly and hepatomegaly; no other abnormalities were observed in these modalities. Hypertension (average systolic blood pressure [SYS]: 167 mm Hg) was observed using the oscillometric method. Haematology (Blind information) was normal and serum biochemical measurements showed azotaemia (blood urea nitrogen: 32.5 mg/dL, RI: 9.2–29.2 mg/dL), hyperlipidaemia (triglyceride: 453 mg/dL, RI: 23–149 mg/dL) and elevation of alkaline phosphatase (ALP) levels (133 U/L, RI: 0–89 U/L; Blind information). The adrenocorticotropic hormone (ACTH) stimulation test was performed by measuring the serum cortisol concentrations at baseline (endogenous cortisol concentration [ECC]) and 1‐h after intravenous (IV) injection of tetracosactide (Blind information; poststimulation cortisol concentration [PCC]). The ECC was normal (2.5 µg/dL, RI: 1–6 µg/dL), while the PCC was high (37.9 µg/dL, RI < 20 µg/dL). Based on clinical signs, laboratory tests and imaging tests, we clinically diagnosed a right kidney tumour and hypercortisolism with a left adrenal tumour. No additional hormonal or imaging tests were performed in order to prioritise treatment of symptomatic kidney tumour and to accommodate dog's anxious temperament, which was incompatible with prolonged restraint.

Trilostane (2.1 mg/kg, q24h) was initiated on day 12. Although the dosage of trilostane was titrated up to 6.3 mg/kg, q24h, the clinical signs persisted. On day 36, blood pressure had normalised (SYS: 137 mm Hg) after adjusting the medications, with pimobendan (0.38 mg/kg, q12h), benazepril (0.5 mg/kg, q12h), furosemide (0.5 mg/kg, q12h), spironolactone (1.25 mg/kg, q12h) and hydralazine (1.5 mg/kg, q12h). We planned to perform a right nephroureterectomy, but we were concerned about postoperative comorbidities related to hypercortisolism, such as hypertension, cardiac overload, urinary tract infection, thromboembolic disease (Hoffman et al., 2018) and delayed wound healing. Controlling hypercortisolism prior to nephroureterectomy was one option. Although higher doses of trilostane could have been tried, this might have delayed the surgery to remove the renal tumour, allowing for its progression. Additionally, we considered that concurrent adrenalectomy and nephroureterectomy could potentially increase the complications risk owing to the complexity of the procedure and prolonged surgical time. In contrast, intraoperative ethanol ablation seemed simpler and had a shorter procedure time than adrenalectomy concurrent with nephroureterectomy. Therefore, after discussion with the owner, we decided to perform a concurrent right nephroureterectomy and laparotomic ethanol ablation of the suspected left adrenal tumour instead of adrenalectomy. The preoperative evaluations were performed 10 days prior to surgery and revealed that there were no suspicious findings of metastasis or lymph node enlargements.

On day 46, open midline right nephroureterectomy and intraoperative ethanol ablation of the suspected left adrenal tumour were performed under general anaesthesia. The dog was premedicated with midazolam (0.2 mg/kg, IV) and fentanyl (3 µg/kg, IV). General anaesthesia was induced using propofol (10 mg/kg, IV) and maintained with isoflurane. Epidural anaesthesia was induced with 0.5% bupivacaine. Fentanyl (2–10 µg/kg/h, continuous rate infusions [CRI]), dobutamine (2–5 µg/kg/min, CRI) and rocuronium (0.5–0.75 mg/kg/h, CRI) were adjusted during surgery by an anaesthesiologist. After the right kidney and ureter were excised, absolute ethanol (2 mL; Blind information) was injected into the cranial pole of the LAG using a 26‐gauge needle. The time required to inject ethanol into the tumour was approximately 5 s for each pole. The injection site immediately turned greyish white. No significant bleeding or circulatory anomalies were observed. The injection site was briefly compressed to restrict the slight backflow of ethanol. The caudal pole was then injected with 2 mL of absolute ethanol. The injection site required a few minutes of compression with gelatine sponges (Blind information) to control the mild to moderate bleeding observed at the caudal pole. In total, the LAG was injected with 4 mL (0.8 mL/kg of body weight) absolute ethanol. The injection volume did not exceed 1 mL/kg of body weight, which is generally considered the upper limit for ethanol embolisation in human medicine (Ko et al., 2009; Mason et al., 2000). A few minutes after the procedure, hypertension (SYS: 233 mm Hg), sinus arrhythmia and multiple ventricular premature complexes (VPC) developed; however, the patient recovered spontaneously within several minutes, without intervention. Time–course vital changes were shown in Table 1. The RAG was subjectively atrophic.

**TABLE 1:** Time–course changes of vital signs during ethanol ablation.

Timing	SYS (mm Hg)	DIA (mm Hg)	MAP (mm Hg)	HR (bpm)
Before nephroureterectomy	159	70	99	87
Before ethanol injection at the cranial pole	149	82	108	75
After ethanol injection at the cranial/before ethanol injection at the caudal pole	182	90	117	89
After ethanol injection at the caudal pole	Not recorded	Not recorded	160	86
5 min after last injection	233	108	140	85
10 min after last injection	144	78	97	98

Blood pressures were measured by oscillometric method.

Abbreviations: DIA, diastolic blood pressure; HR, heart rate; MAP, mean blood pressure; SYS, systolic blood pressure.

On the day after surgery, anorexia and frequent mucoid stools were observed. Serum biochemistry revealed elevated lipase (>1000 U/L, RI: 10–160 U/L) and creatinine (1.59 mg/dL, RI: 0.40–1.40 mg/dL) levels and exacerbation of the azotaemia (75.1 mg/dL). The ECC was sufficient (5.7 µg/dL); thus, the patient did not require glucocorticoid supplementation. A presumptive diagnosis of pancreatitis was made, and as such, an IV infusion (lactated Ringer's solution, 2–3 mL/kg/h) was maintained, and maropitant (1 mg/kg, IV) and fuzapladib sodium (0.4 mg/kg, IV) were added to the treatment regimen. The patient's appetite and stool gradually improved; the patient was discharged 4 days after surgery. The pimobendan (0.38 mg/kg, q12h), benazepril (0.5 mg/kg, q12h), spironolactone (1.25 mg/kg, q12h) and hydralazine (1.5 mg/kg, q12h) were continued at home.

**FIGURE 1:**
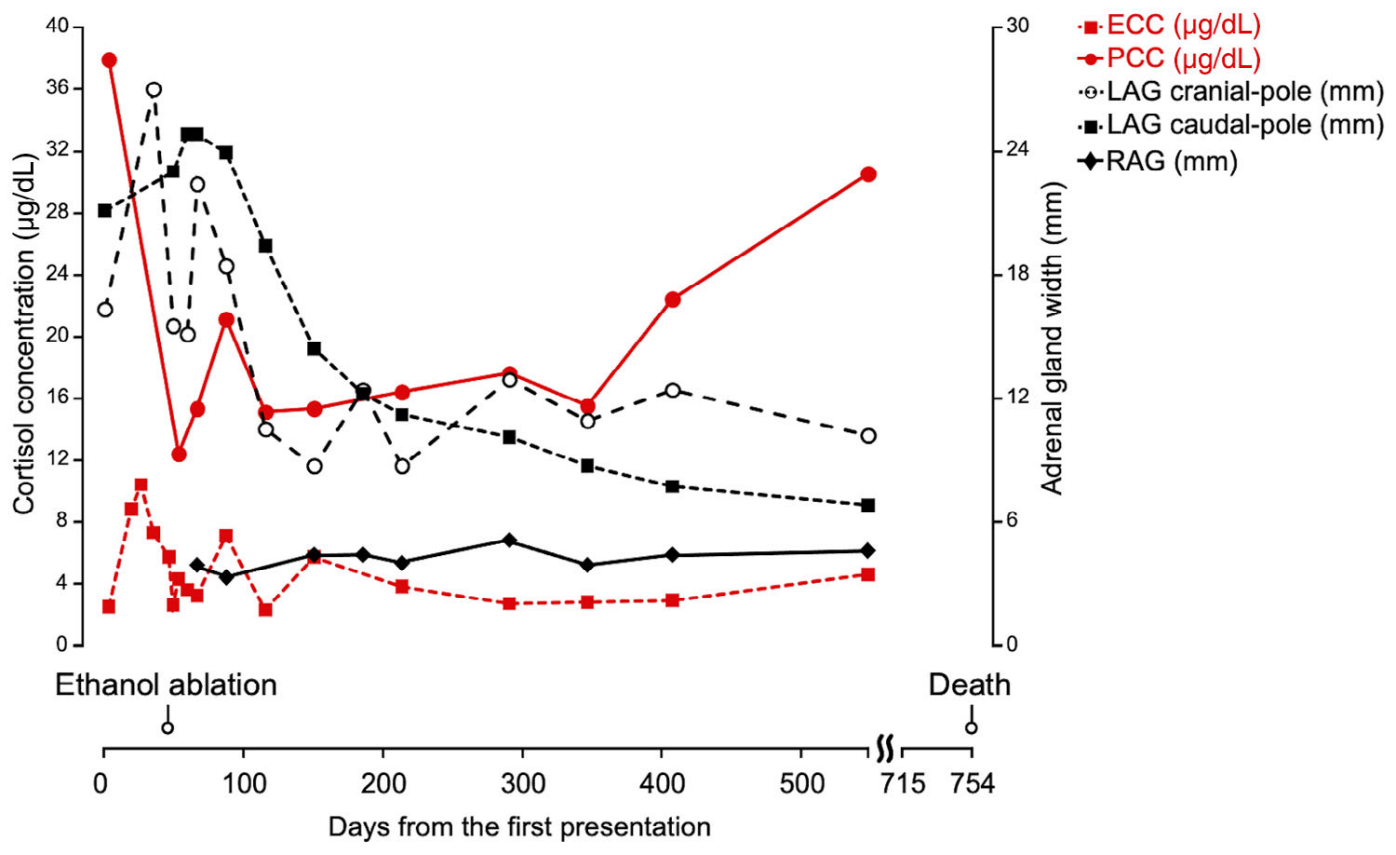
Time–course changes in serum cortisol concentrations (µg/dL) and maximum width of the adrenal gland (mm). The dashed red line represents endogenous cortisol concentration (ECC). The solid red line represents adrenocorticotropic hormone (ACTH) stimulated serum cortisol concentrations (poststimulation cortisol concentration [PCC]). The dashed black lines represent the cranial (white round) or caudal (black square) pole of the left adrenal gland (LAG) width. The solid black line represents the right adrenal gland (RAG) width.

**FIGURE 2:**
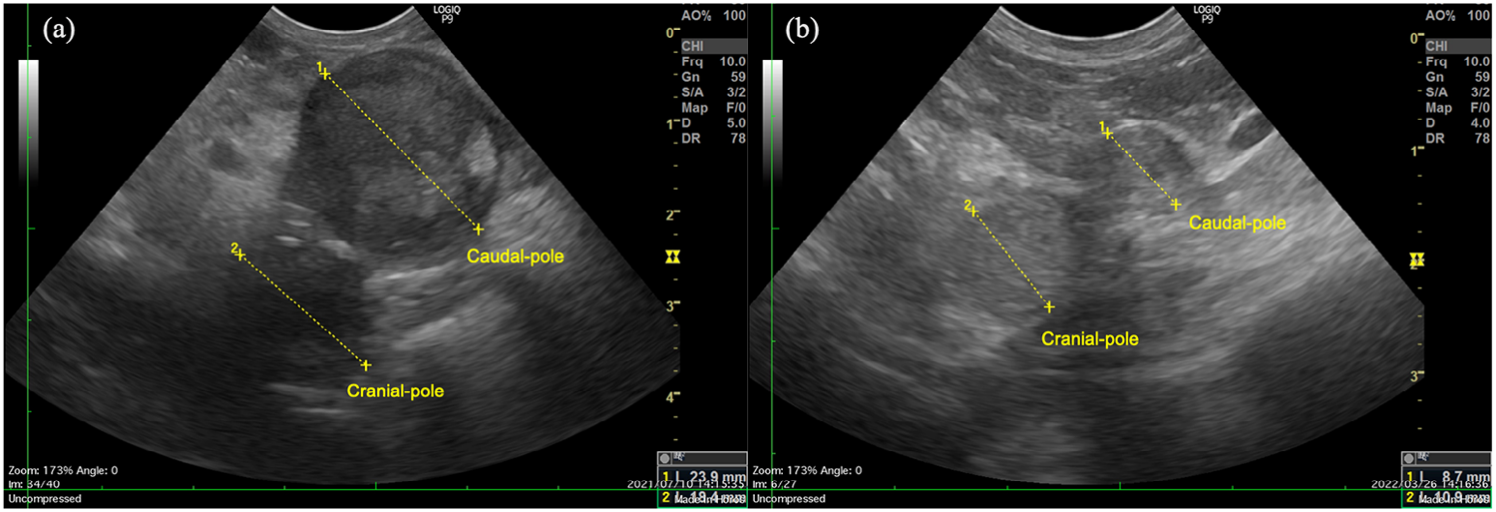
Ultrasonographic images of the left adrenal gland (LAG) over time. (a) Day 88 and (b) day 347 from the first presentation, respectively.

After discharge, the patient's condition improved, and the polyuria/polydipsia and the haematuria resolved. The histopathological diagnosis of the resected right kidney was renal cell carcinoma. The adrenal gland size and cortisol concentrations were measured in a follow‐up (Figure 1). The adrenal gland size was measured using US by the same experienced veterinarian (Blind information). The maximum diameter of the LAG in both the cranial and caudal poles gradually decreased 2.5 months after the procedure (Figure 2). The maximum width of the RAG measured 3.9 mm at 3 weeks postoperatively, which remained within the upper limit of the RI (maximum width 5.3 mm) throughout the follow‐up period. The PCC decreased promptly, 8 days after ethanol ablation, and remained low around the RI for 1 year after the procedure, even without trilostane administration. Additionally, the ECC tended to decrease but were within the RI limit (the mean ECCs before and after the ethanol ablation were 7.25 µg/dL and 3.95 µg/dL, respectively).

On day 242, a 21.7 mm liver mass was found in the caudate lobe. On day 547, ECC was 4.6 µg/dL and PCC was 30.5 µg/dL, suspecting hypercortisolism recurrence; however, there was no change in LAG or RAG size, and no clinical signs. For the convenience of the owner, the next follow‐up was conducted 22 months after the ablation using urine samples. The urinary cortisol/creatinine ratio was in the RI (1.96, RI <1.98; Blind information). The urinary normetanephrine/creatinine ratio was also within the RI (84, RI: 7–124; Blind information). Based on these tests and the absent of clinical signs such as tachycardia or syncope episode, we considered pheochromocytoma unlikely.

On day 750, the patient presented with acute vomiting and diarrhoea. Serum biochemistry revealed severe azotaemia (>140 mg/dL), high serum creatinine (4.61 mg/dL) and phosphate (>15 mg/dL, RI: 1.9–5.0 mg/dL) concentrations. The patient was diagnosed with acute exacerbation of chronic kidney disease due to dehydration and was treated with infusion of lactated Ringer's solution, antiemetics and antidiarrheals. Despite this supportive care, the patient did not improve and died on day 753.

**FIGURE 3:**
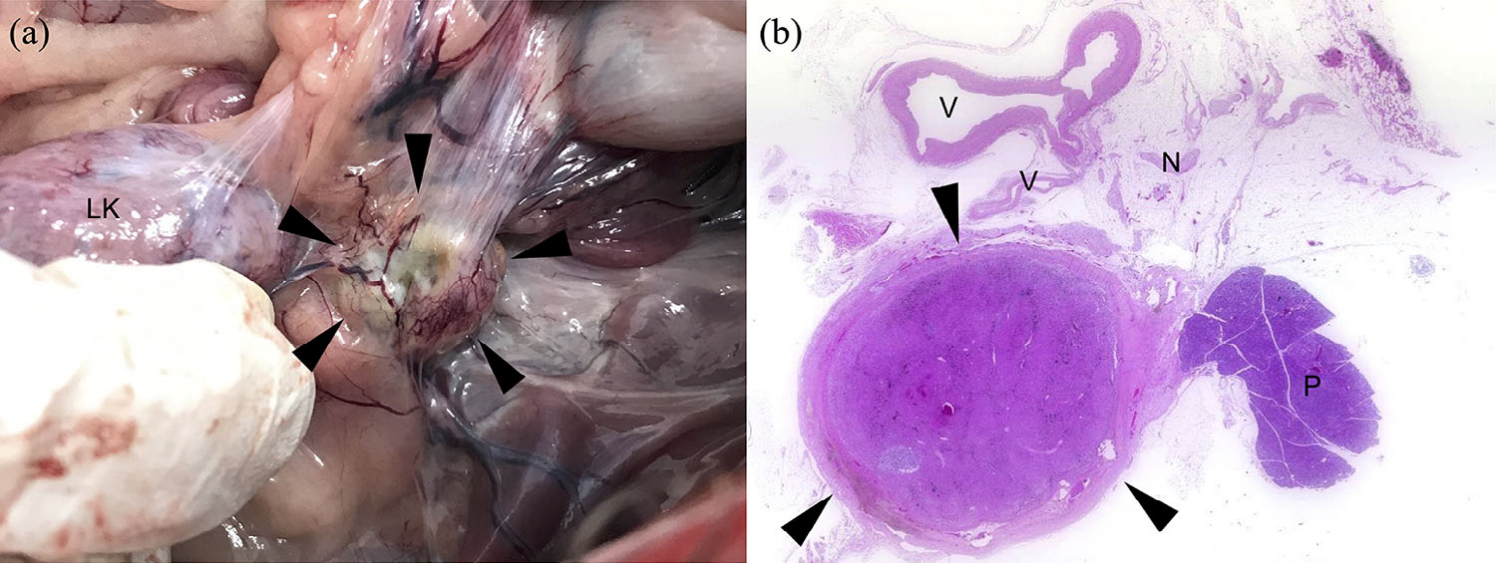
(a) Gross appearance of the left adrenal gland (LAG) on post‐mortem examination. The LAG was trapped in the firm greenish adipose tissue (arrowheads). LK, left kidney. (b) Loupe image of the adrenocortical adenoma in the LAG. Neoplastic tissue was encapsulated by a thick fibrous capsule (arrowheads). The adjacent vessels (V), peripheral nerves (N) and pancreas (P) were intact. Haematoxylin and eosin staining.

Post‐mortem examination revealed that the LAG was surrounded by firm greenish adipose tissue and remained shrunken (Figure 3a). In contrast, the RAG was enlarged compared to its size at the time of surgery. A dumbbell‐shaped nodule was attached to the caudate lobe of the liver. Histologically, the LAG and RAG and the nodule in the liver were diagnosed as adrenocortical tumours (12 mm, 7 mm and 21 mm in diameter in the loupe image, respectively). The ethanol‐injected LAG was encapsulated by a thick fibrous capsule with yellow‐gold pigment and dystrophic calcification. The neoplastic tissue was admixed with haemosiderin‐laden macrophages and extramedullary haematopoiesis, similar to tumours in the RAG and the liver. The adjacent vessels, peripheral nerves and pancreas were intact. No medulla was observed in the LAG (Figure 3b).

## 3 DISCUSSION

We report the well‐controlled hypercortisolism of intraoperative laparotomic ethanol ablation of a left adrenal tumour in a high‐risk surgical canine patient. After the procedure, we observed that the PCC decreased before adrenal tumour shrunk. These results indicate that ethanol ablation impairs the ability of cortisol secretion first and that reduction in tumour size occurs owing to cell death. The tumour regression mechanism of ethanol ablation is considered to be mediated by the dehydrating and protein‐denaturing effects of absolute ethanol, resulting in irreversible protein coagulation and vascular thrombosis of the microvessels supplying tumours (Ellman et al., 1984). In a human retrospective study describing percutaneous chemical ablations of adrenal neoplasms, reduction in tumour size was observed in all 37 patients at 6 months after the procedure (Xiao et al., 2008). Moreover, 84.6% of the primary adrenal neoplasms achieved complete remission at 1 year after the procedure.

ECC tended to decrease after ethanol ablation; however, they were still above the lower limit of the RI, suggesting that ethanol ablation only suppresses excessive cortisol secretion, but does not affect basal cortisol secretion. Indeed, we did not require cortisol supplementation for adrenal insufficiency, a common and life‐threatening complication of adrenalectomy. A systematic review revealed that the mean prevalence of adrenal insufficiency was 99.7% after adrenalectomy in human Cushing's syndrome patients, of which 97.9% underwent unilateral excision (Di Dalmazi et al., 2014). The review also revealed that adrenal insufficiency was temporary, and the patients recovered an average of 11.2 months later (Di Dalmazi et al., 2014). Adrenal insufficiency was also observed in 7.1% of mitotane‐treated and 8.3% of trilostane‐treated dogs with adrenal‐dependent hypercortisolism (Arenas et al., 2014). In contrast, a retrospective study showed no adrenal insufficiency after chemical (ethanol or acetic acid) ablation in human patients with adrenal tumours (Xiao et al., 2008). Temporal adrenal insufficiency has been observed only in bilateral ethanol ablation (Shibata et al., 2000). The biological mechanism involved may be explained by the confinement of topically injected ethanol to relatively small regions around the injection site owing to the low infiltration capacity of absolute ethanol in tumour tissues (Bruix & Sherman, 2005; Shah et al., 2004; Tsai et al., 2008). Postoperative pancreatitis was suspected in this case, but there was no damage to the pancreatic tissue adjacent to injection site. The surgical procedures related to the right nephroureterectomy were possible cause of the pancreatitis.

In the present case, we performed open laparotomic ethanol ablation and had the opportunity to visually observe the procedure. Slight bleeding and ethanol backflow occurred during the procedure. These incidents may be reduced by holding the needles for a few minutes after ethanol injection without withdrawal. Ethanol ablation is not only performed via laparotomy but a percutaneous approach might also be possible. In human medicine, most ethanol ablations of the adrenal gland are performed by CT‐ or US‐guided percutaneous injection (Chang et al., 2012; Frenk et al., 2016; Maki et al., 2000; Rossi et al., 1995; Xiao et al., 2008). In two veterinary studies, dogs with primary hyperparathyroidism were safely treated with US‐guided percutaneous ethanol ablation (Guttin et al., 2015; Rasor et al., 2007).

The patient had transient hypertension, sinus arrhythmia and multiple VPC after ethanol injection, and recovered within a few minutes. Similar complications have been reported previously. A small‐scale retrospective study focusing on CT‐guided percutaneous ethanol ablation of the adrenal glands in humans with hyperfunctioning adrenal disorders revealed that 46% of patients developed major complications, including tachycardia, hyper‐ and hypotension, hypoxemia, acute myocardial infarction and neurological signs; however, no procedure‐related deaths were observed (Frenk et al., 2016). Furthermore, one case report described blood catecholamine level transitions in a hypertensive crisis in a human patient undergoing radiofrequency ablation for metastatic colon carcinoma in the liver (Onik et al., 2003). According to the report, a massive increase in blood catecholamine levels occurred concurrently with a hypertensive crisis when radiofrequency ablation was applied to the right lobe of the liver. Blood catecholamine levels returned to the normal range, and the patient remained normotensive postoperatively. The authors concluded that unintended heat stimulation of the adjacent RAG might have caused the massive catecholamine release, resulting in a hypertensive crisis. In this case, ethanol ablation may have stimulated the adrenal gland, particularly the medulla, inducing catecholamine release and leading to transient hypertension and arrhythmia. In fact, the left adrenal medulla was not observed, suggesting that the medulla may have been ablated by the ethanol.

These complications and their possible mechanisms prompted us to draw the following conclusions. First, when performing ethanol ablation of the adrenal gland, we should prepare ready‐to‐use drugs such as direct‐acting vasodilators and short‐acting adrenergic antagonists with careful monitoring of blood pressure and electrocardiography to deal with the hypertensive crisis (Chini et al., 2004). Second, it is imperative to thoroughly assess the viability of ethanol ablation as a treatment option for functional pheochromocytoma, considering the potential high risk of catecholamine release associated with the procedure, as indicated by previous research findings. In a small‐scale retrospective study, 60% of all major complications occurred in pheochromocytoma; moreover, these complications required intensive care for up to 1 day owing to severe hypertension (Frenk et al., 2016). Contrastingly, a retrospective study on chemical (ethanol or acetic acid) ablation to the adrenal gland in humans with adrenal tumours showed no major complications such as cardiovascular events (Xiao et al., 2008). Notably, this study did not include patients with functional pheochromocytoma because of concerns regarding hypertensive crises. Evaluation of urinary normetanephrine levels, which can be used to diagnose pheochromocytoma (Quante et al., 2010; Salesov et al., 2015; Sasaki et al., 2021), could be useful in reducing the risk of serious complications associated with catecholamine release. However, further studies are needed to evaluate the safety and risks of ethanol ablation for each tumour type.

There was no evidence of RAG enlargement or LAG tumour regrowth, but the PCCs gradually increased from the 13th postoperative month. Simultaneously, US revealed a mass in the liver, which was histologically diagnosed as adrenocortical tumour on post‐mortem examination. This relatively large adrenocortical tumour may have contributed to the PCC elevation. Although the exact origin of the adrenocortical tumour in the liver was unclear because it is difficult to distinguish malignancy in adrenal tumours histologically, several possible scenarios were considered: if adrenal tumour was a carcinoma, the liver lesion was metastasised from it; if it was an adenoma, the liver lesion represented an ectopic adrenal neoplastic transformation. Autopsy findings of the LAG indicated successful local tumour control over the patient's lifetime, suggesting that a single ethanol ablation is a viable treatment for late‐stage elderly patients.

Our study has some limitations. First, we did not optimise the method of ethanol ablation to the adrenal gland. Some researchers have proposed formulas to determine ethanol injection volume in human medicine (Lin et al., 2005; Ohnishi et al., 1998; Xiao et al., 2008); however, this has not yet been done in veterinary medicine. We did not measure blood ethanol concentrations before and after ethanol ablation; thus, the pharmacokinetics and safe dose of ethanol are still unknown. Second, pituitary involvement was unclear because post‐mortem brain tissue was not collected. However, the RAG was subjectively atrophic during laparotomy; therefore, pituitary‐dependent hypercortisolism was clinically unlikely at that time.

## 4 CONCLUSION

Laparotomic ethanol ablation for functional adrenal tumours may be a feasible alternative to conventional adrenalectomy and medical treatments for high‐risk surgical candidates. In addition, ethanol ablation may have advantages, such as a persisting effect, a simple process, less invasiveness and a lower risk of postoperative adrenal insufficiency. However, further optimisation of this procedure is required.

## AUTHOR CONTRIBUTIONS

Shimon Furusato and Eriko Kondo analysed and interpreted the data and wrote the first draft. Eriko Kondo contributed to clinical case management and data collection. Yu Tamura contributed to ultrasonographic examination and provided essential suggestions for the manuscript. Yu Tsuyama critically revised and managed the entire manuscript. All reviewers reviewed and revised the final draft.

## CONFLICT OF INTEREST STATEMENT

The authors declare no conflicts of interest.

### ETHICS STATEMENT

The authors confirm that ethical policies of the journal have been adhered to. All procedures were performed in owned dogs. Informed consent was obtained, and the owners approved the case management and procedures. Additional approval was not obtained for this study.

### PEER REVIEW

The peer review history for this article is available at https://publons.com/publon/10.1002/vms3.70020.

## Data Availability

The data that support the findings of this study are available on request from the corresponding author. The data are not publicly available due to privacy or ethical restrictions.
